# Inhibition Mechanism and Model of an Angiotensin I-Converting Enzyme (ACE)-Inhibitory Hexapeptide from Yeast (*Saccharomyces cerevisiae*)

**DOI:** 10.1371/journal.pone.0037077

**Published:** 2012-05-14

**Authors:** He Ni, Lin Li, Guang Liu, Song-Qing Hu

**Affiliations:** Guangdong Province Key Laboratory for Green Processing of Natural Products and Product Safety, College of Light Industry and Food Sciences, South China University of Technology, Guangzhou, Guangdong, China; Royal College of Surgeons, Ireland

## Abstract

Angiotensin I-converting enzyme (ACE) has an important function in blood pressure regulation. ACE-inhibitory peptides can lower blood pressure by inhibiting ACE activity. Based on the sequence of an ACE-inhibitory hexapeptide (TPTQQS) purified from yeast, enzyme kinetics experiments, isothermal titration calorimetry (ITC), and a docking simulation were performed. The hexapeptide was found to inhibit ACE in a non-competitive manner, as supported by the structural model. The hexapeptide bound to ACE via interactions of the N-terminal Thr1, Thr3, and Gln4 residues with the residues on the lid structure of ACE, and the C-terminal Ser6 attracted the zinc ion, which is vital for ACE catalysis. The displacement of the zinc ion from the active site resulted in the inhibition of ACE activity. The structural model based on the docking simulation was supported by experiments in which the peptide was modified. This study provides a new inhibitory mechanism of ACE by a peptide which broads our knowledge for drug designing against enzyme targets.

## Introduction

Angiotensin I-converting enzyme (ACE, E.C. 3.4.15.1) is a zinc-dependent dipeptidyl carboxypeptidase that plays an important role in regulating blood pressure by catalyzing the conversion of the inactive form of a decapeptide (angiotensin I) into a potent vasoconstrictor, an octapeptide (angiotensin II) [1], [2], [3]. There are two isoforms of ACE in mammals: somatic ACE (sACE), which exists in somatic tissues and is composed of two domains (an N domain and a C domain), and testis ACE (tACE), which is found only in the adult testis. Both isoforms are encoded by the same gene, but the messenger RNAs (mRNAs) transcriptions begin at different positions. The two isoforms have a high level of identity in the C domain, but sACE contains a unique 36-residue sequence at its N terminus [4]. Some studies have shown that the C domain is more important for blood pressure regulation and completely accounts for the blood pressure regulation activity of sACE. The C domain has a higher catalytic constant for angiotensin I and the non-physiological substrate, hippuryl-histidyl-leucine (HHL) [5].

ACE-inhibitory peptides derived from food proteins have attracted particular attention for their ability to prevent hypertension. Compared with chemosynthetic drugs, peptides derived from food proteins may have reduced toxic effects in humans; therefore, these food-derived peptides could be used as potent functional food additives and represent a healthier and more natural alternative to ACE inhibitor drugs. There is evidence that dietary ACE-inhibitory peptides may be bioavailable. Some ACE-inhibitory peptides have resistance to digestion, ability of intestinal absorption and stability in the blood [6], [7], and could produce an acute blood-pressure-lowering effect by oral administration [6], [8]. After the first ACE-inhibitory peptide was obtained from snake venom [9], many other ACE-inhibitory peptides were discovered in the enzymatic hydrolysates of different food proteins [10]. These food protein sources include casein, whey protein, fish protein, chicken eggs, and wheat germ. The inhibitory activities and sequences of some of these peptides are remarkably different.

Because the primary activity of ACE is to cleave the C-terminal dipeptide of oligopeptide substrates with a wide specificity, the inhibitory activity of ACE-inhibitory peptides is strongly influenced by their C-terminal tripeptide sequence. The most potent ACE inhibitors contain hydrophobic amino acid residues at each of the three C-terminal positions that interact with the subsites S1, S1′, and S2′ at the ACE active site. Many studies have shown that peptides with high ACE-inhibitory activities have tryptophan, phenylalanine, tyrosine, or proline at their C-terminus and branched aliphatic amino acids at the N-terminus, and ACE is known to have little affinity for inhibitors with C-terminal dicarboxylic amino acids (e.g., Glu) [11]. However, these structure-activity correlation studies on ACE-inhibitory peptides are only based on amino acid sequence analysis, and many newly identified peptides with high ACE-inhibitory activities do not fit the model based on these studies. Since 2003, the crystal structures of tACE and some complexes of ACE and its inhibitors have been analyzed, and these structures provide a new and intuitive method to analyze the inhibition mechanism [12], [13], [14]. These crystal structures are based on a competitive inhibition mechanism in which ligands occupy the active site of ACE; indeed, competition between HHL and active site-directed inhibitors is frequently utilized to design and modify ACE inhibitors to increase their inhibitory activity [15].

However, many ACE-inhibitory peptides are not competitive inhibitors of ACE, which may be the reason why these peptides are not in accordance with the results of the previous structure-activity correlation studies on ACE-inhibitory peptides. The precise inhibition mechanisms of ACE-inhibitory peptides are not yet clear, and the relationship between the inhibition kinetics and the crystal structure of ACE has not been fully researched. Further investigations are necessary to elucidate the relationship between the inhibition mechanism and the structure of these peptides.

In a previous study [16], we purified a highly active ACE-inhibitory hexapeptide from yeast, Thr-Pro-Thr-Gln-Gln-Ser (TPTQQS); however, this peptide does not have the characteristics of the highly active ACE-inhibitory peptides that have been previous characterized [11]. In this study, we investigated the inhibition mechanism of the hexapeptide on the basis of its sequence to provide a reference for the design of drugs that target such enzymes as ACE.

## Results

### Inhibitory kinetics of an ACE-inhibitory peptide from yeast

**Figure 1:**
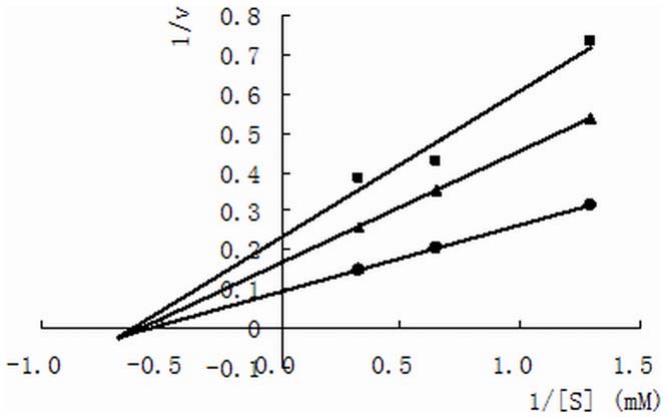
Lineweaver–Burk plot of ACE activity in the presence of the hexapeptide. Control (•), 100 µg/mL of the hexapeptide (▴), and 200 µg/mL of the hexapeptide (▪).

To determine the inhibitory mechanism, Lineweaver-Burk plots were determined for the ACE-inhibitory peptide from yeast (TPTQQS). As shown in Fig. 1, the lines intersection at the 1/[s] axis, indicate that the peptide is a non-competitive inhibitor [17]. Thus, the peptide can combine with the ACE molecule to produce a dead-end complex, regardless of whether a substrate molecule is bound or not. The complex between ACE and TPTQQS can prevent the formation of the reaction product, HA. Competitive inhibition of ACE is more frequently reported, including the common hypertension drugs, such as captopril, enalapril, and lisinopril; these drugs compete with the substrate for binding to the active site of ACE [18]. Some non-competitive inhibitory peptides have also been found in foods such as chickpeas [19], sardines [20], oysters [21], and tuna [22]; however, the inhibition mechanism and the binding site on ACE of these non-competitive inhibitors have not previously been investigated.

### ITC experiments

**Figure 2:**
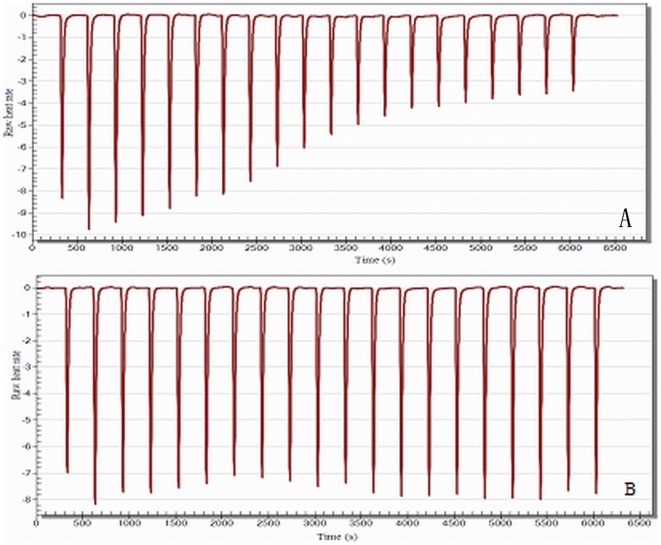
The ITC titration curve. A. Binding of HHL to ACE at pH 8.3. B. Binding of HHL to ACE and TPTQQS at pH 8.3.

Non-competitive inhibition is a system in which the inhibitor and the substrate may both be bound to the enzyme at any given time. When both the substrate and the inhibitor are bound, the enzyme-substrate-inhibitor complex cannot form a product but can only be converted back into the enzyme-substrate complex or the enzyme-inhibitor complex [23]. Thus, ITC experiments were carried out to determine whether TPTQQS is a competitive inhibitor or a non-competitive inhibitor of ACE. As shown in Fig. 2, the titration curves showed a remarkable difference between the addition of HHL to solutions of ACE in the presence or absence of the inhibitor (TPTQQS). In the absence of the inhibitor TPTQQS, the enzymatic reaction showed a typical titration curve (Fig. 2A). When HHL was titrated into the ITC cell, it could bind to ACE and be hydrolyzed to HA by ACE. Initially, the titrated HHL bound to ACE completely, resulting in a higher binding enthalpy (ΔH) and producing a high titration peak. With the increase in the amount of HA, the binding of HHL to ACE was decreased by product inhibition [24], and the reaction tended toward equilibrium. Thus, the peaks corresponding to the raw heat rate gradually decreased in size. Full saturation was achieved at the end of the titration, and background heat of dilution was observed. When TPTQQS bound to ACE in the ITC cell (Fig. 2B), the ITC curve was remarkably different from that for the control experiment in which the peaks for the raw heat rate did not decrease remarkably. In addition, the affinity of HHL for ACE was the same as that in the control experiment, indicating that TPTQQS might act at a different site from the HHL binding site and thus HHL could bind to ACE continuously during the titration experiment [25]. On the basis of these results, we speculated that the binding of TPTQQS to ACE does not prevent the binding of HHL to ACE. Thus, when both HHL and TPTQQS were bound to ACE, the enzyme-substrate-inhibitor complex could not yield HA and could only be converted back into the enzyme-inhibitor complex, allowing another binding event between HHL and ACE. These ITC results are remarkably different from those for a system involving competitive inhibition [26], further verifying that the inhibition mechanism of ACE by TPTQQS is non-competitive.

### The docking of TPTQQS and HHL onto ACE

**Figure 3:**
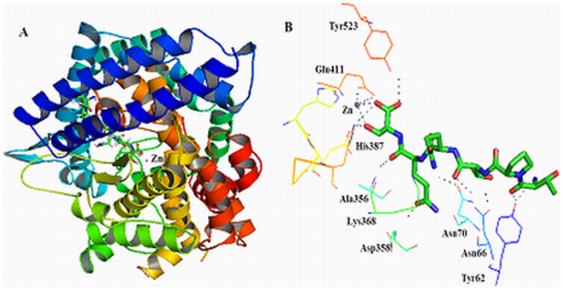
The docking simulation of TPTQQS binding to ACE. A. The docking simulation of TPTQQS (green) binding to ACE (shown as a multi-colored cartoon). A zinc ion (gray) was present in the active site of tACE. B. The interaction between TPTQQS (shown as sticks) and the residues of tACE (shown as lines) is shown.

To investigate the non-competitive inhibition mechanism of ACE by TPTQQS further, DS2.1 (a suitable a program for performing automated docking simulations of ligands to their macromolecular receptors) was used to carry out the docking simulation. Because tACE can completely perform the blood pressure regulation function of sACE, the three-dimensional structure of tACE (1O8A) was used as the macromolecular receptor in the docking simulation to investigate the inhibition mechanism of ACE by TPTQQS [14]. Fig. 3A showed the docking mode of TPTQQS, with the highest LibDock Score (188.439), onto the binding site of ACE. It was observed that this peptide had an expanded conformation and was deeply inserted into the binding pocket. The active cleft divides tACE into two structural subdomains: subdomain I and subdomain II. The peptide was adjacent to subdomain I, far from the active site, which includes the important positions S1′ and S2′, especially [16].

Fig. 3B showed the interaction force between TPTQQS and tACE. The theoretical calculation using PYMOL software indicated that the peptide formed H-bonds with Tyr62, Asn66, Asn70, Ala356, Asp358, Lys368, His387, Glu411, and Tyr523 of tACE [27]. The peptide formed a linker between helix α1 and the zinc ion in the active site. Thr1 formed two H-bonds with Tyr62, Thr3 formed two H-bonds with Asn66, Gln4 formed H-bonds with Asn70 and Lys368, and Gln5 formed H-bonds with Ala356 and Asp358, respectively. These interactions kept the peptide away from the active site of tACE, and only Ser6 of TPTQQS interacted with key amino acid residues in the active site of ACE. Ser6 provided three oxygen atoms to form H-bonds with His387, Glu411, and Tyr523 and form coordination bonds with the zinc ion in the active site of ACE. The distance between the two oxygen ions (Oγ and main-chain carboxyl oxygen ions) of Ser6 and the zinc ion were 1.9 Å and 2.7 Å, respectively.

### The inhibitory activity of the modified peptides from TPTQQS

**Figure 4:**
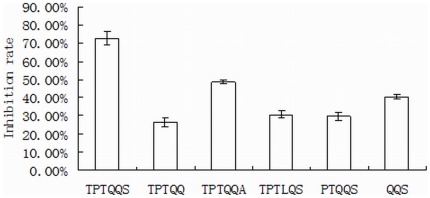
The inhibitory activity of the modified peptides from TPTQQS. The concentration of each peptide was 0.25 mmol/L.

According to the results of the docking of TPTQQS onto ACE, TPTQQS forming coordination bonds with the zinc in the active site and the H-bonds with other amino acids outside of the active site played a key role in the inhibitory effect of TPTQQS. We chemically modified the key amino acids and changed the length of TPTQQS to investigate the non-competitive inhibition mechanism of TPTQQS further. First, we confirmed the effect of Ser6 by deleting the residue and changing it to an alanine in the peptide. The results showed that the inhibition rate of the peptide significantly decreased from 72.55% to 26.36% when the Ser was deleted as a result of the lack of interaction between the Ser and the active site of ACE. When Ser6 was replaced with Ala6, the interaction force decreased because Ala contains only two oxygen ions that can interact with the active site of ACE; the inhibition rate decreased to 48.61% (Fig. 4).

Second, to investigate the effect of the other amino acids in TPTQQS on the inhibitory activity, we deleted one or three residues from the N-terminus and changed Gln4 to Leu4 to weaken the interaction force between TPTQQS and amino acid residues outside of the active site of ACE. When the strength of the interaction between TPTQQS and helix α1 of tACE was decreased, the inhibition rate decreased by more than 40% (Fig. 4). Thus, it was again demonstrated that the H-bonds between TPTQQS and residues outside of the active site of ACE played a key part in the non-competitive inhibition of ACE by TPTQQS.

## Discussion

TPTQQS has been shown to be an effective ACE-inhibitory peptide that was isolated from hydrolyzed yeast protein in our previous study [16]. However, the amino acid sequence of this peptide is not in accordance with the results of structure-activity correlation studies on ACE-inhibitory peptides [11]. However, these studies are based on a competitive inhibition mechanism in which ligands occupy the active site of ACE, interacting with key amino acid residues, such as HEXXH [11]. Therefore, we hypothesized that TPTQQS is a non-competitive inhibitor of ACE. In the present study, we demonstrated that TPTQQS is a non-competitive inhibitor of ACE by performing kinetics and ITC experiments.

**Figure 5:**
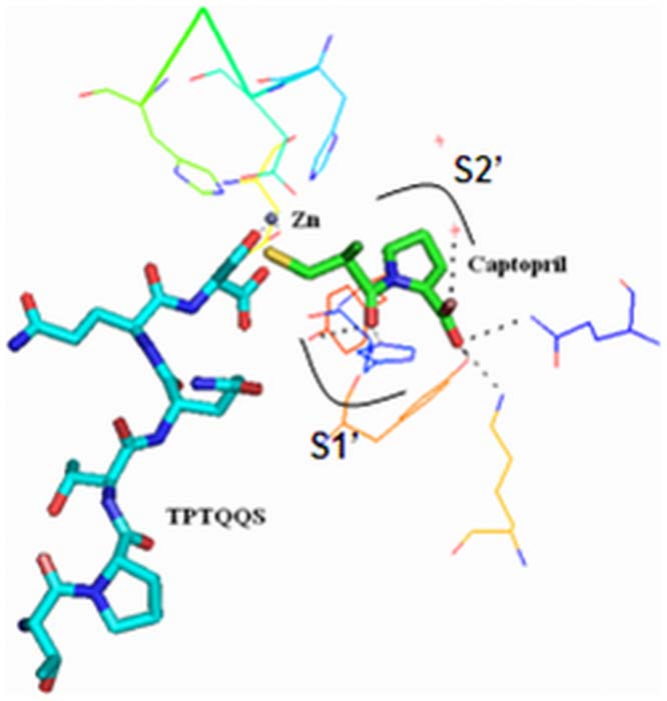
Overlap of captopril (green) in the crystal structure of the captopril-ACE complex with TPTQQS (cyan) in the docking simulation for TPTQQS. The figures were prepared using PYMOL software.

The active site of ACE is composed of a zinc ion and a HEXXH…E motif, which includes His383, Glu384, His387 and Glu411. ACE accommodates two substrate residues that are C-terminal to the zinc ion (in the S1′ and S2′ subsites) and, thus, has dipeptidase activity. The S1′ and S2′ subsites are conserved and important subsites in ACE and are occupied by competitive inhibitors. According to the proposed mechanism of HHL hydrolysis catalyzed by ACE [28], [29] and the crystal structure of the captopril-ACE complex (PDB code 1UZF), as a competitive inhibitor of ACE, captopril forms H-bonds with key residues in the active site of ACE and occupies the key positions that would be occupied by HHL, such as the S1′ and S2′ subsites, consistent with the typical competitive inhibition model (Fig. 5). We found that TPTQQS does not occupy the S1′ and S2′ subsites and is not located in the active site of ACE, only Ser6 can interact with the zinc ion, and the other amino acids are not located near the active site of ACE. Therefore, we concluded that TPTQQS cannot compete with HHL for the active site of ACE, indicating that TPTQQS is a non-competitive inhibitor of ACE structurally.

**Figure 6:**
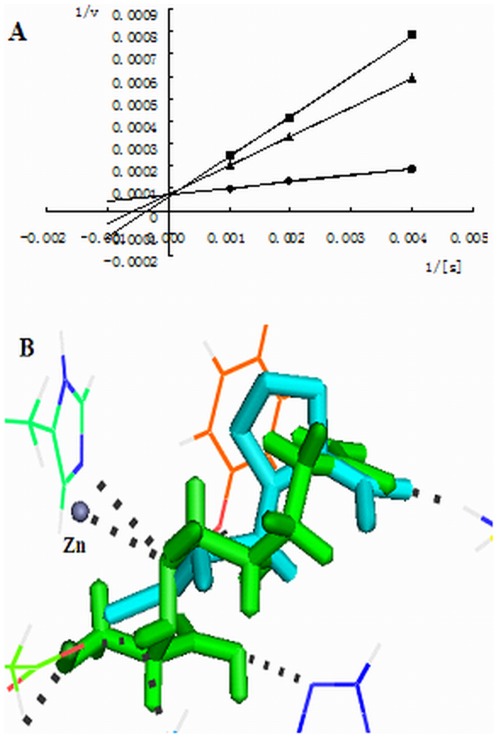
The mode of tACE inhibition by QS. A. Lineweaver–Burk plot of the ACE activity in the presence of the hexapeptide; control (•), 100 µg/mL of QS (▴), and 200 µg/mL of QS (▪). B. The docking simulation of QS (green) binding to ACE (shown as sticks), and the overlap with captopril (cyan) in the crystal structure of the captopril-ACE complex The zinc ion (gray) is shown as nb_spheres. The figures were prepared using PYMOL software.

The docking simulation of TPTQQS binding with ACE showed that Thr1, Thr3 and Gln4 are the key amino acids for the non-competitive inhibition: all of these amino acids interacted with residues in helix α1 of ACE. The three N-terminal helices (helices α1 to α3) form a lid-like structure (referred to as the lid) that has significant flexibility according to the B-factor [30]. Thr1, Thr3 and Gln4 can orient the peptide onto the lid structure, keeping the peptide out of the active site of tACE. The inhibition model was further verified by deleting Thr1, Pro2, Thr3 and Gln4 (Fig. 6). The kinetics experiment and the docking results for QS demonstrated that when the H-bonds between the peptide and the residues outside of the active site in tACE were deleted, the mode of inhibition became competitive, as demonstrated by the kinetics experiment (Fig. 6A). In this scenario, QS occupies the active site (S1′ and S2′) of tACE, which is occupied by the competitive inhibitor, captopril, in the crystal structure (Fig. 6B).

The zinc ion is the key ion in the active site of tACE, and this ion can be moved away from the opposite wall of the activesite cleft by residues 569–578, thus opening up the catalytic site [31]. In the docking simulation, the zinc ion could be attracted by Ser6 through coordination bonds, forcing the zinc ion away from the active site and resulting in a change in the conformation of the active site. Thus, although HHL could bind onto the active site, it could not be catalyzed to form HA because the zinc ion could not coordinate with it tightly.

**Figure 7:**
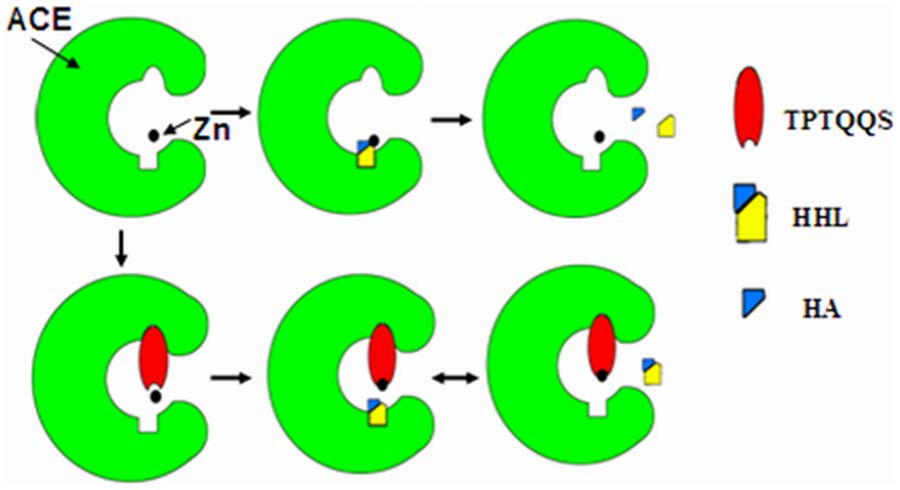
Model of the inhibition of ACE by TPTQQS. The model shows that TPTQQS moves the zinc ion away from the active site to inhibit ACE.

These results suggest that the inhibition of ACE by TPTQQS is non-competitive, and a non-competitive inhibition model of TPTQQS to ACE can be built (Fig. 7). When ACE is in the unbound form, the zinc ion and HEXXH motif compose the complete active site of ACE, and HHL can enter the active site and be converted into HA [29], [32]. After TPTQQS enters ACE, the Thr1, Thr3 and Gln4 residues of TPTQQS allow the peptide to interact with the lid structure of tACE, and the C-terminal Ser6 pulls the zinc ion away from the active site through the coordination bonds between the Ser and the zinc ion, resulting in the non-competitive inhibition of ACE by TPTQQS. Although HHL could bind to the active site, it could not be converted into the product, HA, because the conformation of the active site had changed. This model supports the non-competitive inhibition mechanism. In conclusion, this study provides a new inhibitory model and mechanism of ACE by a peptide which broads our knowledge for drug designing against enzyme targets.

## Materials and Methods

### Assay of ACE-inhibitory activity

The determination of ACE-inhibitory activity was performed using a High Performance Liquid Chromatography (HPLC) method, modified from a spectrophotometric method [33]. HHL (Sigma, USA) was dissolved in 0.1 mol/L Na-borate buffer (pH 8.3) containing 1.55 mmol/L NaCl. ACE (Sigma, USA) was dissolved in the same buffer at a concentration of 0.2 U/mL. A mixture containing 10 µL of inhibitor and 5 µL of ACE solution was incubated at 37°C for 5 min, 50 µL of HHL solution was added, and the solution was incubated for 1 h. The reaction was stopped by adding 100 µL of 0.1% trifluoroacetic acid (TFA) (Sigma, USA). The amount of hippuric acid (HA) yielded by ACE catalysis was measured by RP-HPLC (GX-281, Gilson, France) with an octadecylsilyl (ODS) column (4.6 mm×250 mm, 5 µm, Agilent, USA). The mobile phase was 60% methanol (V/V) containing 0.1% TFA at a flow rate of 0.8 mL/min. The effluent was monitored at 228 nm. The ACE-inhibitory activity was calculated using Equation (1) as follows:
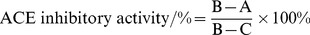
(1)where A is the content of HA generated in the presence of an ACE inhibitor, B is the content of HA generated without an ACE inhibitor, and C is the content of HA generated without ACE.

*Inhibition kinetics of TPTQQS*- Various substrate (HHL) concentrations (0.78 mM, 1.55 mM, and 3.10 mM) were incubated with the ACE solution in the presence or absence of 100 µg/mL, of 200 µg/mL TPTQQS at 37°C. The ACE-inhibitory activity was determined as described as described in the “*Assay of ACE-inhibitory activity*". The kinetics of ACE in the presence of the inhibitory peptide was determined using Lineweaver-Burk plots [34].

### Docking studies

The molecular modeling package, ChemOffice 2004 (Cambridge Scientific Computing, Cambridge, Massachusetts, USA), was used to construct and view the two- and three-dimensional structures of the hexapeptides. The structures of all of the ligands were optimized using “Prepare Ligands" in Discovery Studio (DS) 2.1 (Accelrys Software, Inc., San Diego) [35], [36], [37]. The three-dimensional structure of ACE (PDB code 1O8A) was obtained from the Protein Data Bank [13]. The optimized ligand molecules were docked into the refined protein model using “Dock Ligands (LibDock)" in DS. The relative energy, the absolute energy and the LibDock score were obtained, and the latter was used as the final criterion to identify the best docking mode [38].

### ITC experiments

The isothermal titration calorimetry (ITC) experiments were carried out using a Nano ITC Low Volume System (TA Instruments-Waters LLC., USA) with a 190 µL cell. All of the experiments were performed at 37°C. ACE was prepared by diluting the stock solution in a buffer of the appropriate pH; the substrate was dissolved in the same buffer as the enzyme. In a control experiment, the ITC cell was filled with 0.024 µmol/L ACE (250 µL), and the ACE solution was titrated with 1.55 mmol/L HHL. The injection volume was 50 µL, and the time between two injections was 300 s during which the substrate was completely hydrolyzed. In the inhibition experiment, the ITC cell was filled with 0.024 µmol/L ACE and 0.13 µmol/L TPTQQS (250 µL), and the solution was titrated with 1.55 mmol/L HHL. The titration conditions were the same as those for the control experiment.
